# A primary cardiac schwannoma of the right ventricle: a case report and literature review

**DOI:** 10.1186/s12872-022-02941-x

**Published:** 2022-11-22

**Authors:** Fang Wang, Lin Li, Heng Ma, Xiao-Xiao Chi

**Affiliations:** 1 Department of Radiology, Shangdong Province, The Affiliated Yantai Yuhuangding Hospital of Qingdao University, No. 20, Yuhuangding East Road, Zhifu District, Yantai, 264000 People’s Republic of China; 2 Department of Pathology, Yucheng People’s Hospital, Yucheng, Dezhou, China

**Keywords:** Cardiac tumour, Schwannoma, Case report

## Abstract

**Background:**

Primary cardiac schwannoma remains extremely rare and difficult to distinguish from other myocardial tumours. We report a case of cardiac schwannoma that occurred in the lateral wall of the right ventricle and grew in the myocardial walls. It is the third case of schwannoma that occurred in the free wall of the right ventricle. Moreover, we reviewed and summarised the literature for cases involving benign cardiac schwannomas.

**Case presentation:**

We present a case of a 64-year-old woman who presented to our centre with syncope for 1–2 min. Echocardiogram and contrast-enhanced computed tomography subsequently revealed a 2.9 × 1.9 cm homogeneous mass originating from the anterior wall of the right ventricle. The patient underwent thoracotomy to resect the mass, which was pathologically verified as Schwann cell tumour.

**Conclusions:**

This is a rare case added to the limited existing literature on cardiac schwannoma. Comprehensive analysis of various imaging examinations is helpful to determine the extent of the tumour. Complete surgical resection is recommended for similar cases involving cardiac schwannomas, especially when the patient has related symptoms. Patients generally have a good prognosis. The pathogenesis of cardiac schwannoma needs further research in order to prevent and manage this rare lesion.

## Background

Primary cardiac tumours are extremely rare tumours with a prevalence of 0.02%–0.056% [1], and cardiac Schwann cell tumours are even rarer. Schwannoma is a slow-growing tumour that arises from Schwann cells in the surrounding nerve sheath [2]. Primary cardiac schwannoma is believed to originate from the cardiac plexus or the cardiac branch of the vagus nerve [3]; but its pathogenesis remains unclear. We explored the pathogenesis of cardiac schwannomas that have not been explored in detail in previous literature. Cardiac schwannoma has a variety of clinical manifestations, ranging from asymptomatic findings on imaging studies to exertion, chest pain, tachypnoea and arrhythmia, which are related to tumour size and compression of adjacent structures (e.g. large vessels, cardiac chambers, mediastinal structure and coronary artery [4]). Preoperative diagnosis is difficult, but the identification of such tumours is of great value in the development of treatment strategies and prognostic assessment. In this report, we present a case of a cardiac schwannoma, which is the third case of schwannoma that occurred in the free wall of the right ventricle. In addition, we reviewed and summarised cardiac schwannomas, which have been reported in the English literature. This is the most detailed summary and discussion of cardiac schwannoma in the past 18 years (Table 1).

**Table 1:** Summary of the reported cases of cardiac schwannoma

No	Author	Year	Age(years)	Sex(M/F)	Location	Symptoms	Size(cm)	Imaging findings	Treatment	Survival and prognosis	Follow-up time (mouth)	Comorbidities
1	Hallman et al. [5]	1966	12	F	Anterior RV surface near AV groove	Easy fatigability, dyspnea on exertion	3	Unknown	Unknown	Unknown	Unknown	Unknown
2	Gleason et al. [5]	1972	26	F	RA near its junction with IAS, 2 cm below inlet of SVC	Heart murmur, incidental finding at operation (ASD, PS)	1.5 × 1.5	Unknown	Unknown	No recurrence	Unknown	Unknown
3	Stephen Factor et al. [6]	1976	55	F	Lateral border of RA, superior to AV groove	Incidental finding at autopsy	7 × 5.5 × 5	–	–	–	–	Crystadenocarcinoma of the ovary, hypertension, intermittent intestinal obstruction
4	Betancourt et al. [7]	1979	32	F	Intracavitary tumor attached to parietal band of crista	Chest pain, shortness of breath	8.75 × 6.25	Marked cardiomegaly on chest X-ray	Surgery with CPB	No recurrence	36	Hypertension
5	Monroe et al. [5]	1984	70	M	Anterior LV surface, below AV groove	Incidental finding at autopsy	7	Unknown	–	–	–	Lung cancer
6	Andrew D. Forbes et al. [8]	1994	35	M	Posterior LA between inferior PV and CS	Exertion-related paroxysmal atrial fibrillation, atypical chest pain	4 × 7	Unknown	Median sternotomy + CPB	No recurrence	6	None
7	Kodama et al. [5]	1995	50	M	Anterior RA, superior to AV groove	Exertional dyspnea	9 × 5 × 6	Unknown	Unknown	Unknown	Unknown	Unknown
8	Hashimoto T et al. [9]	1998	46	F	Between SVC and ascending Ao	None	12 × 8 × 7	Cardiomegaly on chest radiograph	Median sternotomy + CPB	No recurrence	24	Uterine fibroids
9	Bizzarri et al. [3]	2001	72	M	Intracavitary tumor attached to floor of RA, close to AV	Shortness of breath, chest pain	5 × 4 × 4	Unknown	Surgery with CPB	Recovered quickly	None	Mild hypertension, right renal adenocarcinoma
10	Mustafa Sirlak et al. [10]	2003	61	F	LA	Shortness of breath,atrial fibrillation with a 10-year duration	9.5 × 8.5 × 6.5	Heterogeneous and hypodense mass with central cystic foci	Median sternotomy + CPB	Remains well and disease-free	2	None
11	Kunihide Nakamura et al. [5]	2003	33	F	Anterior RA extending LA and PV	None	5 × 5.2 × 4.5	Inhomogeneous enhancement	Median sternotomy + CPB	No recurrence	12	None
12	Davinder S. Jassal et al. [11]	2003	49	F	RA adjacent to the AV groove	Mitral valve prolapse presented with pleuritic chest discomfort	6.4 × 5.5 × 3.4	Large heterogeneous mass	Surgery with CPB	Unknown	Unknown	None
13	Xiao-dong chen et al. [12]	2005	51	F	RA	Dizziness, tinnitus, gait instability	10.2 × 10	Heterogeneous mass with calcifications and Cystic structure inside	Median sternotomy + CPB	Unknown	Unknown	Bilateral acoustic neuroma, type II neurofibromatosis
14	T. Rausche et al. [13]	2006	42	F	RV epicardium	Persistent coughing	11 × 7	Mass with areas of different echodensities	Median sternotomy	Completely inconspicious	Unknown	None
15	Noedir A. G. Stolf et al. [14]	2006	56	F	RA, close to the cavo-atrial junction	None	6.0 × 4.8	Heterogeneous solid tumoral mas with calcifications inside	Surgery with CPB	No recurrence	36	Cavernous mass of the bladder
16	Serdar Sevimli et al. [15]	2007	57	F	The free wall of the LV	Palpitations	5.5 × 6	Containing cystic structures	Surgery with CPB	Remained disease free	3	None
17	Saverio La Francesca et al. [4]	2007	30	F	Anterior and lateral surface of the superior half of the LV	None	4 × 4 × 9	A large multilobed cardiac mass	CPB + coronary reconstruction + thrombectomy + LVAD + anticoagulation	Discharged home on postoperative day 28	None	Cancer of the left chest wall
18	Sarah A Early et al. [16]	2007	57	M	Posterolateral wall of the RA extending to the interatrial septum	No cardiovascular symptoms	4.3 × 5.2	Heterogenous very mild enhancement on MRI	Surgery with CPB	Excellent	Unknown	Gastritis, normochromic normocytic anaemia
19	Corey D et al.[17]	2011	67	M	RA involving the interatrial septum	Dyspnea on exertion and syncope	3.1 × 2.5 × 1.7	Intraoperative transesophageal echocardiogramrevealed a cystic mass	Surgery with antegrade cardioplegia	Do well after his surgery with no symptoms	9	Severe aortic stenosis
20	Kristen Elstner et al.[18]	2013	65	M	Lateral wall of the LPA	Dyspnoea on exertion	5.2 × 4.5 × 4.1	Heterogeneous enhancement	Median sternotomy + CPB + CABG	No signs or symptoms	Unknown	None
21	Su Kyung Hwang et al.[19]	2014	55	F	LA,attached to the left atrial appendage	Chest pain at rest	4.3 × 4 × 3	Mass with hemorrhagic formation and a pericardial tail	Median sternotomy + CPB	No remnant mass	12	None
22	Kuk Hui Son et al.[20]	2015	42	F	Atrial roof between the aorta and the SVC	Palpitations on several occasions	10 × 9.5	Heterogeneously enhanced	Sternotomy + CPB + 3D printing model	Discharged without relevant complication	None	None
23	Joon Chul Jung et al.[21]	2015	69	F	Interatrial septum	None	2.8 × 2.7 × 2.5	Cystic mass,broad base	median sternotomy	Recovered without problems	Unknown	Sigmoid colon cancer
24	Ji-Gang Wang et al.[22]	2018	59	M	RA, attached to the underpart of interatrial septum	None	4.5 × 3.5 × 3	Unknown	Unknown	No recurrence	Unknown	None
25	Zhixiong Huang et al.[23]	2020	53	M	Behind the ascending Ao	Dyspnea on exertion, hypertension	8.2 × 7.1 × 6.9	Cystic low density mass	Median sternotomy + CPB	No recurrence	60	Hypertension
26	Kenji Yokoyama et al.[5]	2021	46	M	Posterior wall of the LA	None	1.4 × 1.6	Multiple lesions(posterior mediastinum, left pulmonary hilar area)	Median sternotomy + CPB	No recurrence	12	Type II neurofibromatosis
27	Wang SYet al.[24]	2021	65	F	RA adjacent to atrial septum	Shortness of breath after activity	–	Apparent FDG uptake in the mass, SUV-max: 5.2	–	Unknown	Unknown	Unknown
28	Present case	2021	64	F	Anterior RV	None	2.8 × 2.0	Shallowly divided, homogeneous,	Median sternotomy + CPB	No recurrence	60	Lung adenocarcinoma

*RV* Right ventricle, *AV* Atrioventricular, *RA* Right atrium, *IAS* Interatrial septum, *SVC* Superior vena cava, *ASD* Atrial septal defect, *PS* Pulmonary stenosis, *CPB* Cardiopulmonary bypass, *LV* Left ventricle, *LA* Left atrium, *PV* Pulmonary vein, *CS* Coronary sinus, *LVAD* CentriMag left ventricular assist device, *MRI* Magnetic resonance imaging, *LPA* Left pulmonary artery, *CABG* Coronary artery bypass surgery, *Ao* Aorta

## Case presentation

A 64-year-old woman was admitted to our institute with syncope for 1–2 min. She reported no shortness of breath, chest pain, dyspnoea or weight loss. Her medical history included lacunar infarction and ground glass nodule of the left upper lobe, which was suspected to be lung adenocarcinoma. On physical examination, the patient was afebrile and had a regular heart rate of 60 beats per minute, a blood pressure of 125/70 mmHg and a respiratory rate of 16 breaths per minute. The patient had no murmur. The results of laboratory investigations were unremarkable. Electrocardiogram revealed sinus bradycardia and a ventricular rate of 57 beats per minute.

**Fig. 1:**
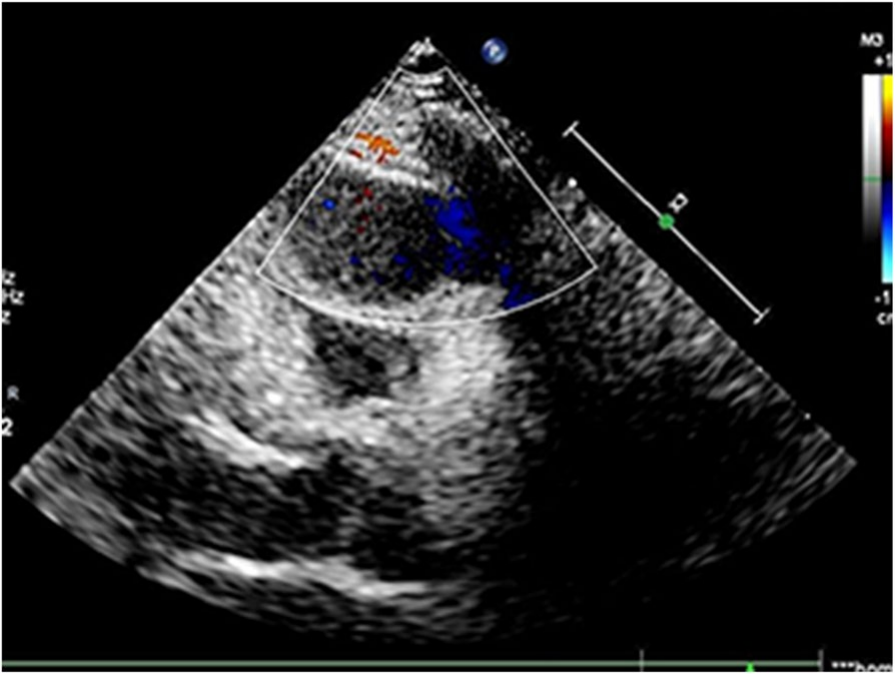
A hypoechoic mass on the anterior wall of the right ventricle with uniform internal echo, shallow lobules, star-like blood flow signals, clear boundaries and a size of approximately 2.9 × 1.9 cm

**Fig. 2:**
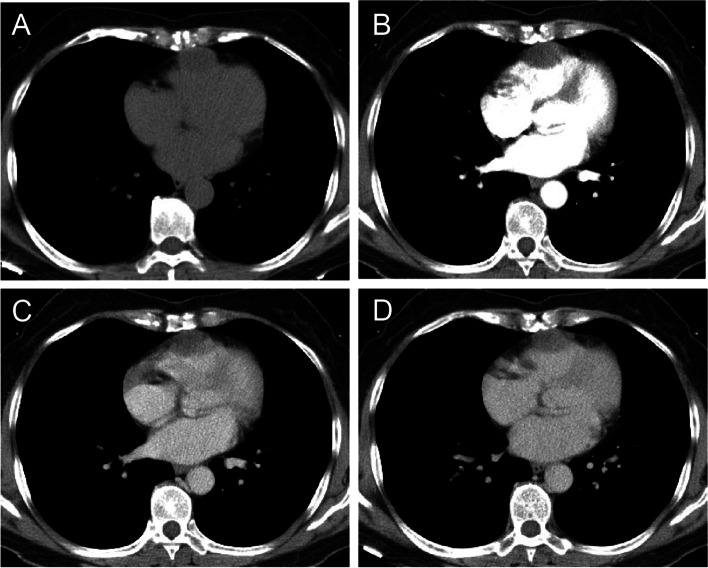
**A** An isodense mass on the anterior wall of the right ventricle with uniform internal density and clear boundary. No obvious narrowing of the heart cavity was notede, the CT value is approximately 29 HU. **B** A myocardial tissue mass with slightly enhancement during the arterial phase, the CT value is approximately 34 HU. **C-D** Lesions with a persistent enhancement during the venous and delayed phase, the CT value is approximately 38 HU (**C**) and 35HU (**D**)

Echocardiogram revealed a mass with a size of 2.9 × 1.9 cm on the front wall of the right ventricle, which had a uniform internal echo and star-shaped blood flow signals (Fig. 1). Chest computed tomography (CT) scan demonstrated a 2.8 × 2.0 cm homogeneous mass originating from the anterior wall of the right ventricle, which has a relatively broad base. The boundary of the mass is clear and fixed. No obvious narrowing of the heart cavity was observed (Fig. 2A). The patient underwent a three-phase dynamic chest CT, which disclosed a myocardial tissue mass with slightly enhancement during the arterial phase (Fig. 2B) and a persistent moderate increase during the venous and delayed phases (Fig. 2C-D). The definite diagnosis was difficult. The patient was referred to thoracic surgery for thoracotomy and resection of the myocardial tumour under cardiopulmonary bypass (CPB). The defect of the right ventricle was repaired.

**Fig. 3:**
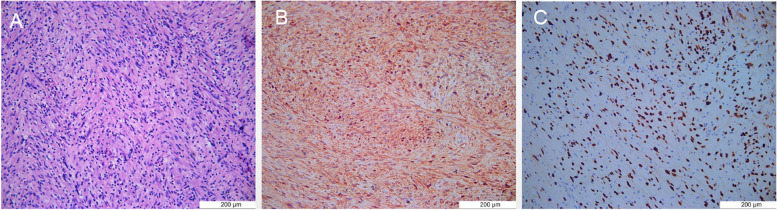
**A** Cells of Antoni A tissue have modest eosinophilic cytoplasm with on discernible cell borders and normochromic, elongated,tapered nuclei. (Hematoxylin and eosin) **B** Tumour cells stained positively for S100 as determined by immunohistochemistry.(S100) **C** Tumour cells stained positively for SOX10 as determined by immunohistochemistry.(SOX10)

The desquamated tissue was histopathologically examined and reported as a schwannoma. Microscopic sections revealed that Cells of Antoni A tissue have modest eosinophilic cytoplasm with on discernible cell borders and normochromic, elongated, tapered nuclei (Fig. 3A). Immunohistochemical studies showed that the tumour cells stained positively for S100 and SOX10 (Fig. 3B-C). Micrographs were acquired by using Nikon CI2 (Nikon) and NIS-Elements (Nikon) software. The resolution of each acquired images is 300 dots per inch.

The patient recovered and was discharged on the 10th day after surgery without complications. The patient had a ground glass nodule resection, which was subsequently confirmed as microinvasive lung adenocarcinoma, and the pathologic TNM stage was T1N0M0. The patient recovered uneventfully and had no sign of recurrence at a follow-up duration of 5 years.

## Discussion and conclusion

**Fig. 4:**
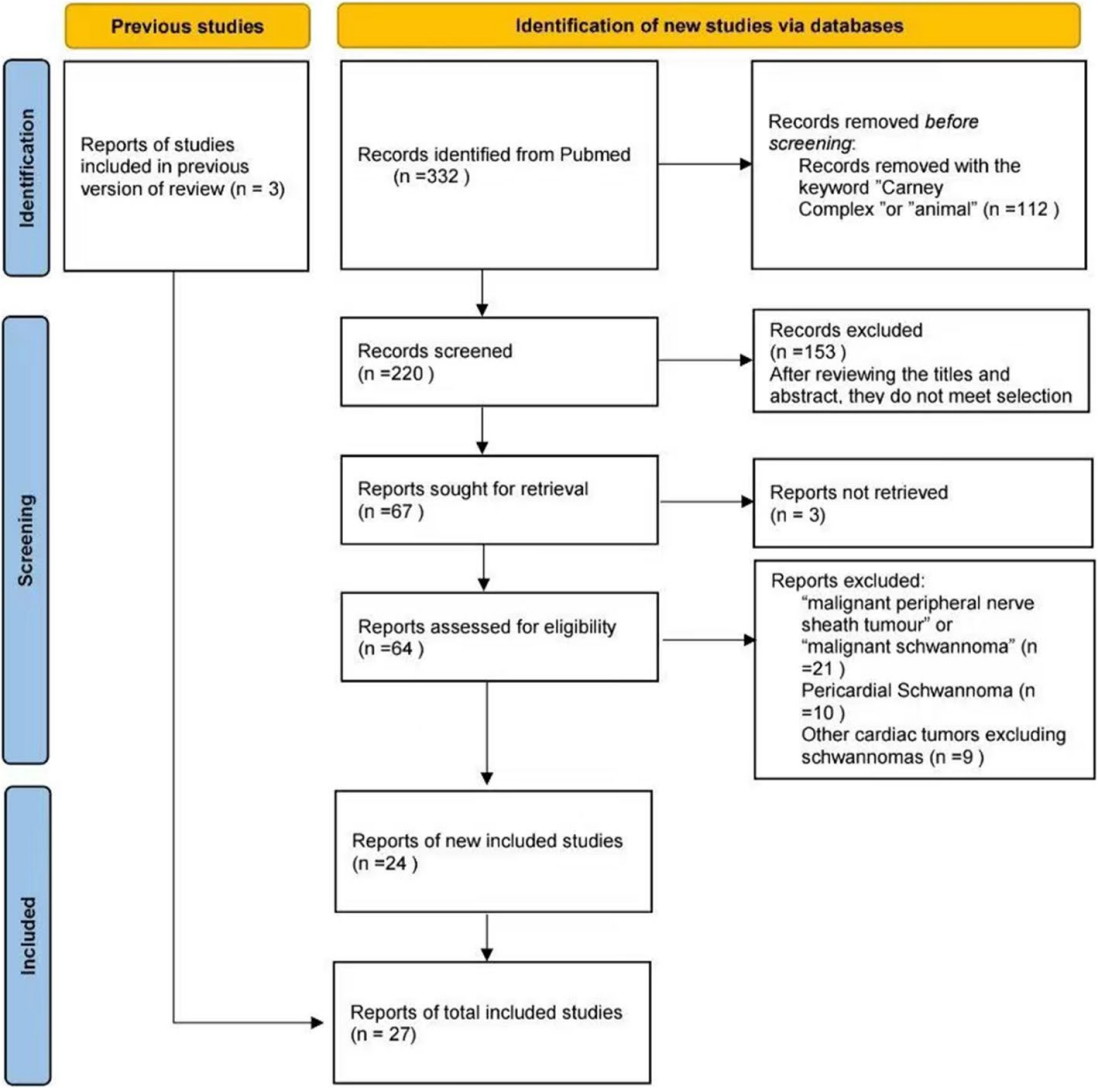
Selection of studies for inclusion

We searched the PubMed database until July 21 2022 using the keywords “Cardiac Schwannoma” and “Cardiac tumour and Schwannoma” to identify the relevant English medical literature. The search identified 332 results. After a careful analysis of the articles, approximately 24 articles met the inclusion criteria and were included. In addition, 3 patients dates from one of the abovementioned paper [5], which could not be retrieved from PubMed, were also added to this review. The study selection process is shown in Fig. 4. Two reviewers independently appraised all included studies using the Joanna Briggs Institute (JBI) checklist for case reports and case series.

Primary cardiac tumours are very rare, and benign cardiac schwannomas are even rarer. Our review of English literature showed that 27 cases of benign cardiac schwannomas including two cases of type II neurofibromatosis [12, 25] have been reported. The age range was 12–72 years old, the mean age was 50.7 years old, and the male-to-female ratio was about 1:2. These data were consistent with previous reports [5]. Primary cardiac schwannoma is believed to originate from the cardiac plexus or the cardiac branch of the vagus nerve; therefore, it is located primarily on the right side of the heart [26]. However, we found that the right atrium is the predominant site of cardiac schwannomas (12/28), and the incidences of left atrial, bilateral ventricular and aortic outflow tracts have no remarkable differences. This finding could be attributed to the distribution of the sinoatrial and atrioventricular nodes around the right atrium because the distribution of nerve fibres around these structures is remarkably higher than that in the surrounding working myocardium. The case that we reported occurred in the lateral wall of the right ventricle. It is the third case of schwannoma that occurred in the free wall of the right ventricle. This study provides an important supplement to explore the pathogenesis of the lesion and reflect the distribution of cardiac plexus.

Neurilemoma originates from the peripheral nerve sheath, and its pathogenesis remains unclear. No relevant literature has proposed hypotheses regarding its cause. The National Toxicology Program and Ramazzini Institute reported that radiofrequency electromagnetic field substantially increases glioma and schwannoma in the heart of rodents [27]. Stephen Factor et al. reported that a patient with cardiac neurilemoma who received a large total amount of radiotherapy or at least one course of radiotherapy directed to the lower thoracic vertebral region for the treatment of paravertebral mass may have peripherally involved the heart [6]. The relationship between human cardiac schwannoma and radiation needs further research. Additionally, Das Gupta et a1. studied 303 benign schwannomas and reported the interesting correlation of nerve sheath tumours with the past, concurrent or future development of a malignancy unrelated to peripheral nerves [28]. Through case review, we found that seven cases, including our case, were accompanied by other tumours, including six cases of malignancy and one unspecified case. The seven tumours included one autopsies case of ovarian cancer [6] and one autopsies cases of lung cancer [5], one case of renal cancer preceded cardiac schwannoma [3], one case of synchronous sigmoid colon cancer [21], one case of synchronous cancer of the left chest wall [4] and one case of synchronous cavernous mass of the bladder [14], one cases of synchronous lung adenocarcinoma (our case). The connection between schwannoma and other unrelated malignancy needs further experimental verification.

Primary cardiac schwannomas vary in size. The clinical symptoms are mostly caused by compression or obstruction, and some patients may have dyspnoea on exertion (5/28) [12, 17, 18, 23], chest pain (4/28) [3, 7, 8, 19], shortness of breath (4/28) [3, 7, 10, 24], palpitation (2/28) [15, 20], arrhythmia (2/28) [8, 10] and other discomfort. More than one third of patients (10/28) [4–6, 9, 14, 16, 21–23] had no related symptoms. Our case was hospitalised because of syncope, which is rarely reported in literature. The syncope may be caused by the sudden decrease or pause of cardiac output caused by the cardiac tumour.

Cardiac schwannoma can be detected by X-ray or echocardiogram, CT and magnetic resonance imaging (MRI), which can help to better determine the location and extent of the mass and the involvement of other structures [3]. Tumours are mostly heterogeneous masses with cystic changes, haemorrhages and calcifications. Uneven and mild enhancement may even occur. Some lesions have a broad base and shallow lobes, and most lesions have a clear boundary. The fibrous capsule is also one of the identification points of schwannomas from other tumours. Coronary angiography is required for patients at risk of coronary heart disease or with tumours that may involve the coronary artery [4]. When the exact origin of the tumour cannot be obtained by CT or MRI, 3D printing and model establishment can help to clearly identify the location of the tumour and its relationship with large blood vessels [20]. The nature of the tumour is difficult to identify through imaging.

Most patients with cardiac Schwann cell tumours undergo extensive radical tumour resection and cardiac reconstruction with autologous pericardium or artificial patch under CPB [5]. The degree of involvement and reconstruction of the atrioventricular valve, coronary artery, coronary sinus or pulmonary vein are also important [4, 5, 13]. Among the 28 patients, excepting for 2 autopsy patients, 2 patients whose survival/death was not mentioned in the literature, and 1 patient whose data was not available, the survival rate of the remaining 23 patients was 100% in the follow-up period, and the postoperative prognosis is good. Our operation was also successful, and no recurrence was observed after 5 years of follow-up.

In conclusion, this is a rare case added to the limited existing literature on cardiac schwannoma. Comprehensive analysis of various imaging examinations is helpful to determine the extent of the tumour. Complete surgical resection is recommended for similar cases involving cardiac schwannomas, especially when the patient has related symptoms. Patients generally have a good prognosis. The pathogenesis of cardiac schwannoma needs further research in order to prevent and manage this rare lesion.

## Data Availability

All data generated or analysed during this study are included in this published article and its supplementary information files.

## Abbreviations

CT: Computed tomography
CPB: Cardiopulmonary bypass
MRI: Magnetic resonance imaging
